# Comparison of Colorimetric Methods for Measuring the Solubility of Legume Proteins

**DOI:** 10.3390/gels10090551

**Published:** 2024-08-25

**Authors:** Terrence Dent, Allison LeMinh, Farnaz Maleky

**Affiliations:** Department of Food Science and Technology, The Ohio State University, 2015 Fyffe Court, Columbus, OH 43210, USA

**Keywords:** protein solubility, calorimetric methods, measurement accuracy, gelation

## Abstract

Increasing the use of plant proteins in foods requires improving their physical and chemical properties, such as emulsification, gelation capacity, and thermal stability. These properties determine the acceptability and functionality of food products. Higher protein solubility significantly impacts these properties by affecting denaturation and the stability of emulsifiers or gels. Therefore, developing plant-based protein ingredients requires accurately and conveniently measuring their solubility. Colorimetric solubility methods overcome many issues of more robust combustion and titration methods, but complicated chemical mechanisms limit their applicability for certain proteins. This study aims to compare the effectiveness of four common colorimetric solubility measurement methods for pulse and non-pulse legume proteins and hydrolysates. Pea, chickpea, lentil, and soy protein isolates were made from defatted flour and their solubility at a range of pHs was measured using the Bradford, Lowry, bicinchoninic acid (BCA), and biuret methods. Solubility was also measured for chickpea and soy protein hydrolysates made using Alcalase and Flavourzyme. A comparison of the methods for solubility quantification revealed that the Bradford and Lowry methods most closely match the expected results for the unhydrolyzed protein, with the BCA and biuret methods underestimating solubility by 30%. The Lowry method was the preferred method for hydrolysate solubility measurement, with the Bradford method measuring 0% solubility at the isoelectric point due to an inability to interact with peptides that are soluble at this pH. This study identifies reliable methods for measuring plant protein solubility that establish uniform outcomes and enable a better comparison across studies, giving a consensus for key functional properties in food applications.

## 1. Introduction

In recent years, there has been an increasing demand for plant-based proteins from consumers. From a survey of 1000 random people, grocery sales of plant-based meat replacers grew 12.1% from July 2020 to July 2021 [1]. In addition, there has been a 43% increase in U.S. households that have purchased alternative meat products, such as plant-based products. These trends are driven by consumers’ desire to eat healthier and more ethically [1].

For plant-based proteins to be suitable for food products, they must possess desirable functional characteristics like foaming, gelation, emulsification, water-binding capacity, solubility, interfacial properties, and lipid interactions. The efficacy of these functional attributes depends significantly on the solubility of the protein ingredient. Solubility serves as a key indicator for understanding protein–water interactions, including surface charge, surface hydrophobicity, and other characteristics like particle size that influence their overall functionality in food applications [2]. Specifically, studies have documented a positive correlation between protein solubility and their gelation capacity. Gels stabilize food products by holding water and other components in a structured matrix, which prevents separation and extends shelf life. Understanding and accurately measuring protein solubility under different pH levels is vital for effective gelation, as pH influences protein charge, solubility, and the interactions necessary for forming a stable gel network. This knowledge allows food scientists to optimize gelation conditions, ensuring consistent product quality and enabling the development of innovative food products with tailored textures and functionalities.

Early research has introduced a number of established, non-colorimetric methods to determine protein solubility, including the Dumas and Kjeldahl methods, which are the current industrial standards. These methods are chosen for their reliability in quantifying protein content, essential for ensuring product quality and consistency in various industries. Both methods are based on measuring the nitrogen content in the supernatant of a centrifuged protein solution in water and comparing that to the total nitrogen content of the solution. The Dumas method is combustion-based and has its advantages, as analysis is fast and no few chemical reagents are used. The problem, however, is that the method uses expensive equipment, which does not measure the true protein value [3]. The Kjeldahl method utilizes a strong acid and titration steps requiring hazardous chemicals that produce waste. The Kjeldahl method is also time-consuming and does not measure the true protein value [3]. Dumas and Kjeldahl require conversion factors to calculate nitrogen content. The conversion factors can be inaccurate or inconsistent, as asparagine and glutamine residues are responsible for differing nitrogen conversion factors between different proteins [2]. To obtain accurate values, it is necessary to follow additional experiments and steps to have a specific nitrogen conversion factor. Considering the aforementioned drawbacks, many researchers prefer to use colorimetric methods to measure a protein’s solubility.

Past research efforts have introduced various colorimetric methods, such as the Bradford, biuret, Lowry, and bicinchoninic acid (BCA) methods. These methods all rely on some form of interaction between a coloring agent and protein to produce colored complexes that can be quantified using light absorbance. The Bradford method is a dye-binding method where the Coomassie Brilliant Blue G-250 interacts with proteins. Coomassie comes in a doubly protonated form in which it has a reddish-brown color [4]. The dye donates a proton to the exposed hydrophobic pocket of the protein, which binds the dye to the protein. In the bound, deprotonated form, Coomassie G-250 becomes blue with a max absorbance at 595 nm [4]. The biuret method is a copper-ion-based method. The biuret method is based on proteins with two or more peptide bonds that form complexes with cupric ions [4]. The reaction mixture turns to violet color, and the reduced copper is detected at 540 nm [3].

The Lowry and BCA were developed to improve upon the biuret method [4]. The Lowry method is a combination of the biuret method with the reduction of the Folin–Ciocalteu phenol reagent. This reduction occurs upon reactions with tyrosine and tryptophan residues on the proteins after hydrolysis, and the reduced form of the reagent is a bluish color [3,4]. Depending on the protein concentration and sensitivity desired, absorbance is measured at 750 nm or 500 nm (absorbance at 750 nm is for a higher sensitivity and low protein concentration) [4]. The BCA method is based on a principle similar to that of biuret; proteins and peptides reduce cupric ions to cuprous ions [3,4]. In BCA, the cupric ions react with bicinchoninic acid (a green reagent) and form a purple complex for measuring the absorbance at 562 nm [4]. The peptide bonds, as well as cysteine, cystine, tryptophan, and tyrosine residues, are responsible for the reduction of cupric ions [2,3,4].

Although there are many advantages to using colorimetric methods for solubility determination, they have some drawbacks depending on the protein’s type and structure. For example, bovine serum albumin (BSA), used for creating a standard curve in these methods, may not be applicable for legume proteins. The protein ribulose 1,5-diphosphate carboxylase-oxygenase (RUPD) has been identified as a potential replacement for BSA when determining the solubility of plant-based proteins as it is the most abundant protein in many plants (Jones et al., 1989) [5]. The addition of polyvinylpyrrolidone (PVP) has also been shown to improve plant-based protein solubility determination (Jones et al., 1989) [5]. In addition, colorimetric methods are unable to account for chemical differences between proteins, which may add to the inconsistency of the measurements. For instance, these cannot be used for comparison of soy and legumes proteins because soy is structurally/chemically different from pulses (soy protein consists of a majority of 11S and 7S with a minor fraction of 2S proteins, which differ from pea protein) [6]. Additionally, there are significant differences in the amino acid composition of different plant-based proteins and these are sensitive to non-protein impurities, which further contributes to variability in solubility measurements (Gorissen et al., 2018) [7].

The importance of accurate solubility measurements becomes more complicated with protein hydrolysis, as this process significantly modifies protein structure and physical properties. Hydrolysis is an additional step for plant proteins to improve their functionality, which affects the proteins’ primary sequence, structure, and size of peptides. Hydrolysis is known to affect the solubility of proteins, particularly near their isoelectric point (pI) Wouters et al., 2016) [8,9,10]. The drawbacks and time-consuming nature of the established solubility methods in combination with the complexities of plant-based proteins necessitate the identification of a colorimetric solubility method that is faster and provides reliable results. Given the variability in solubility methods and protein properties, this study postulates that not all common colorimetric solubility methods will yield accurate results for plant protein solubility. Thus, this study aims to determine which commonly used colorimetric solubility method reliably produces expected solubility results for plant-based proteins at a range of pHs. This study uses legume proteins as well as hydrolysates made with Alcalase and Flavourzyme to test the accuracy of solubility methods for native and hydrolyzed protein isolates.

## 2. Results and Discussion

Measuring solubility at a range of pHs is common in solubility studies to simulate solubility in a range of food product environments. Studies have shown that most proteins, including the legume proteins used in this study, follow a U-shaped pH–solubility curve with a minimum solubility value near their isoelectric points and higher values at extreme acidic and basic pH values by plotting the protein solubility vs. pH from pH 2 to 8 [11,12]. It is also known that the isoelectric points of plant-based proteins vary widely based on their amino acid composition, with most legume proteins having an isoelectric point between pH 4 and 5 [12,13,14]. The U shape of the solubility curves can be accentuated when protein isolates are isolated via isoelectric precipitation due to selectively purifying proteins insoluble at pH 4 to 5. The isolation process also preferentially isolates globulin proteins by eliminating proteins soluble at the isolation pH (e.g., albumins) [15].

**Figure 1:**
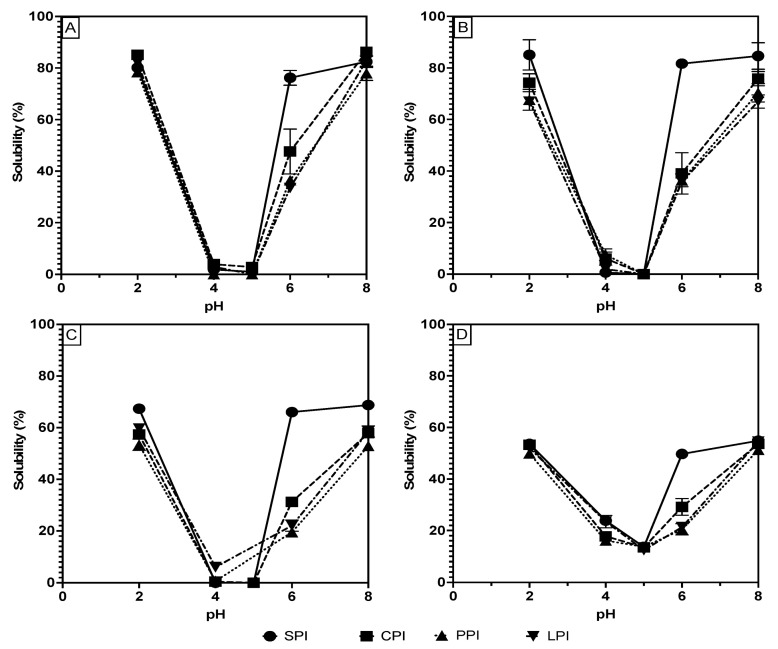
pH solubility curves of soy, chickpea, pea, and lentil proteins measure using the Bradford (**A**), Lowry (**B**), BCA (**C**) and biuret (**D**) methods.

Therefore, in this study, protein solubility at various pHs (2, 4, 5, 6, and 8) was measured and plotted, as shown in Figure 1A–D, to compare the data obtained from each measurement technique and to thoroughly assess the solubility of the extracted and processed proteins. Figure 1 generally demonstrates that the expected U-shaped solubility character is preserved for all proteins and under all measuring techniques. There are, however, significant differences in the solubility values of all the samples when comparing the methods. Table 1 shows that, at pH 2, the proteins’ solubility values are generally highest for Bradford (ranges of 78.4 to 85.1%), followed by Lowry (ranges of 66.9–85.0%), then BCA and biuret (53.3–67.3% and 49.8–53.6%, respectively). A similar trend was observed at the opposite extreme pH (pH 8), where Bradford shows the significantly (*p* < 0.05) highest solubility, followed by Lowry, BCA, and biuret. The values range from 77.9 to 86.3% with the Bradford method, 66.9 to 84.6% for Lowry, 52.9 to 68.7% for BCA, and 51.3 to 54.8% for biuret. At pH 4 and 5, the solubility of all proteins under all methods except for biuret was less than 10%. The low sensitivity of the biuret method may account for its flattened solubility curve and less extreme solubility values at pH where high and low solubility are expected.

Considering the proteins’ isolation process, one may expect 100% solubility at the extreme pH values; however, less than 100% solubility of the samples is likely attributable to the contribution of non-protein impurities to the protein mass, as well as losses of solubility due to denaturation during processing [16]. The loss of solubility attributable to processing can be assumed to be low; however, the isolation process that caused the least amount of functionality loss was chosen [16]. Therefore, Bradford and Lowry are likely accurate solubility measurements because the high measured solubilities match the expected high solubilities at the extreme pH values. Conversely, the BCA and biuret solubility measurements in Table 1 are shown to be significantly lower than the Bradford and Lowry measurements in every case, thus bringing into question their effectiveness for use in plant-based protein ingredient analysis. The biuret method is known to have a low sensitivity, thus potentially explaining the lower protein solubility measurements given the same protein concentrations as the other methods [17]. The biuret method is also known to be sensitive to the association state of proteins as it requires complexation on two non-continuous peptide bonds to form the colored complex [18]. Considering that most legume proteins are composed of globulins that associate in a higher-order quaternary structure, this may explain the low sensitivity to biuret complex formation [15,19]. Similar to biuret, the BCA method relies on the reduction of cupric ions followed by complexation with the BCA reagent. This method is known to be particularly sensitive to impurities and variations between protein composition [18]. Overall, considering that BSA is used as the standard for these solubility measurements, the difference between standard and sample protein composition may result in differences in colored complex formation [7]. These differences can lead to incompatibility of the standard curve with legume proteins, thus making the results inaccurate.

In addition to solubility variation among the methods, Figure 1 and Table 1 also document solubility differences among pea, chickpea, and lentil proteins compared to soy at pH 6. At this pH, soy proteins showed a significantly higher range of 49.7–81.6% than chickpea (29.1–47.6%,), pea (20.4–36.4%,), and lentil (21.3–35.8%). These differences can be explained by the proteins’ amino acid composition and their classifications. Pea, chickpea, and lentil are classified as pulses, while soy is not, and this categorization partially reflects their amino acid composition [20]. These differences in composition manifest as charge differences at pH 6, resulting in lower charge-based repulsion and higher intermolecular interaction in the pulse proteins at pH 6 and therefore lower solubility. Hence, pulse proteins may exhibit different solubility behavior at intermediate pH, which may change their potential applications in food products.

Hydrolyzed proteins are often utilized in the food industry, but their differing chemistry and structure from unhydrolyzed proteins may alter their compatibility with certain solubility measurement methods. Specifically, the fact that solubility decreases after hydrolysis has been observed in numerous studies, and current research suggests that the formation of peptides during hydrolysis leads to protein–protein interactions and the formation of insoluble aggregates [21,22,23].

To study solubility methods on legume protein hydrolysate, a subset of the previously used proteins and methods was narrowed down to assess solubility methods for hydrolysates. Chickpea (a representative for the pulse proteins, such as pea and lentil) and soy protein (a non-pulse legume with different solubility range) were used to assess hydrolyzed protein solubility. The Bradford and Lowry methods were shown, in this study, to be the most accurate and viable methods for unhydrolyzed proteins; so, they were also analyzed for effectiveness on hydrolysates. BCA was also used to evaluate hydrolysate solubility, as it is very similar in mechanism to the biuret method but was shown to be more accurate than biuret. Additionally, two different types of enzymes, and endo- and exo-protease, Flavourzyme and Alcalase, respectively, were selected for the differing length of peptides that are formed during hydrolysis [16,24].

**Figure 2:**
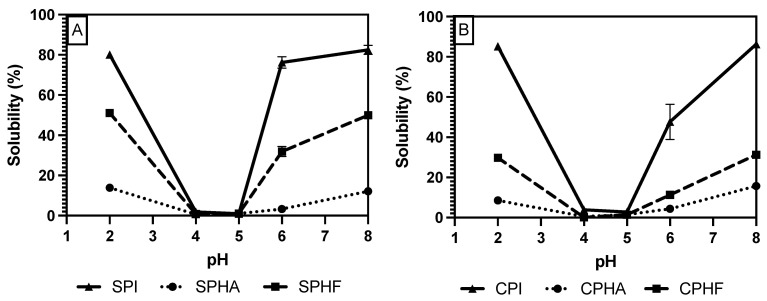
pH solubility curves of soy (**A**) and chickpea (**B**) isolates and hydrolysates measured using the Bradford method.

**Figure 3:**
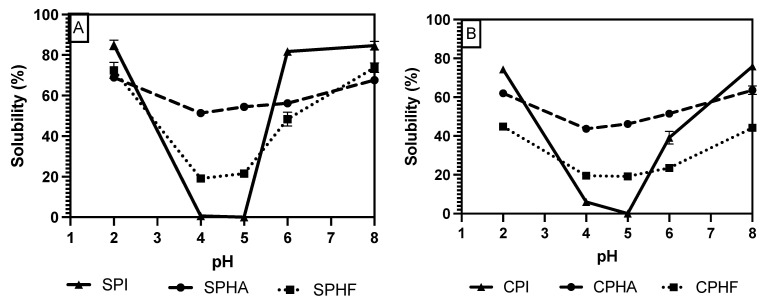
pH solubility curves of soy (**A**) and chickpea (**B**) isolates and hydrolysates measured using the Lowry method.

**Figure 4:**
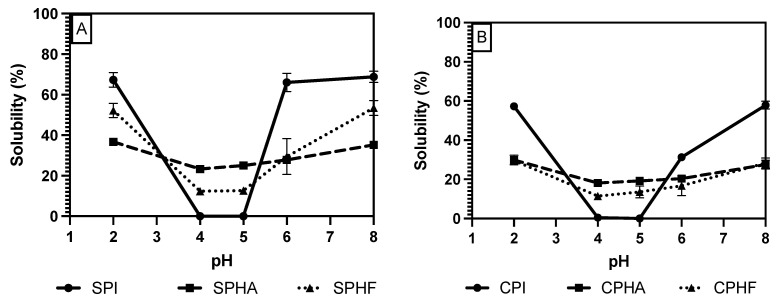
pH solubility curves of soy (**A**) and chickpea (**B**) isolates and hydrolysates measured using the BCA method.

Comparing solubility differences between unhydrolyzed and hydrolyzed proteins, Figure 2, Figure 3 and Figure 4 show a modest reduction in solubility at extreme pH for hydrolyzed proteins compared to unhydrolyzed for all enzyme–protein systems and an elevated solubility around the isoelectric point, in the case of Lowry and BCA. For example, as shown in Table 1 and Table 2, chickpea isolate has a solubility of 74.2% with the Lowry method at pH 2, while the Alcalase and Flavourzyme hydrolysates show 62% and 44.8% solubility, respectively. However, a comparison of hydrolysate solubility between methods shows differing results and pH solubility curve shapes. As shown in Figure 2, Figure 3 and Figure 4 and Table 2, hydrolysate solubility is significantly (*p* < 0.05) higher using the Lowry method for every pH condition and protein. For example, at pH 2, the soy–Alcalase’s solubility was measured at 68.9% with the Lowry method, but 36.6% and 13.9% for BCA and Bradford, respectively. More importantly, the Lowry method shows the highest solubilities at pH 4 and 5 when comparing hydrolysates of the same type with moderate solubility with BCA and virtually no solubility with the Bradford method. Notably, the Lowry and BCA solubility curves do not show the typical U-shaped solubility curve of unhydrolyzed proteins, while Bradford does. Most studies agree that the solubility of hydrolysates is higher at the isoelectric point compared to unhydrolyzed isolates due to the formation of peptides with isoelectric points different from those of intact globular proteins, thus changing from a U-shaped to a flat solubility curve [25,26]. These elevated solubility measurements near the pI indicate that the Lowry method is most effective for hydrolysates as it is able to quantify the presence of soluble peptides. The Lowry method is the logical choice for hydrolyzed proteins based on the results of this study. This method includes a hydrolysis step with 1 N NaOH at 100 °C for 10 min, which frees tryptophan and tyrosine residues for redox reactions with copper ions [24,27].

The deviation from the expected results from the Bradford method may be explained by the mechanism of Coomassie binding to proteins. Coomassie binds to proteins in its neutral form, causing a shift in absorbance maxima which is proportional to protein concentration. The binding of the dye is mediated by association to positively charged amino acids, as well as hydrophobic interactions [18,28]. The importance of van der Waals interactions and hydrophobic pockets in dye binding may explain the unexpected results in Bradford measurements of hydrolysate solubility [29,30]. Hydrolysis results in the release of short peptides, thus disrupting the structure of native proteins and cleaving hydrophobic peptides [31]. This exposure of hydrophobic peptides may interfere with the structure of hydrophobic pockets where Coomassie binds, and therefore lead to minimized protein–dye complex formation. The lowered solubility of BCA compared to Lowry observed in this study, especially near the pI, can be partially explained by differences in protein composition between the legume proteins studied and the BSA standard used. BCA is sensitive to differences in protein composition and relies on interactions between peptide bonds and copper ions to produce colored complexes [18]. The application of hydrolysis reduced the number of peptide bonds and may therefore make this method further incompatible with the unhydrolyzed BSA standard that is used.

The emerging usage of plant-based proteins in commercial food products requires simple and fast methods for protein solubility quantification. The comparison of the Bradford, biuret, Lowry, and BCA methods reveals that the Bradford and Lowry methods are more accurate for unhydrolyzed proteins, while the Lowry method is most suited for hydrolyzed proteins. An understanding of these recommendations could facilitate a unification of methods in future research in the plant-based protein field, thus improving accuracy and the ability to compare results more confidently between studies.

## 3. Conclusions

The measurement of solubility across a range of pH values is a common practice in solubility studies to mimic various food product environments. In this study, protein solubility at different pH levels was measured to thoroughly assess extracted and processed proteins. While all proteins exhibited the expected U-shaped solubility curve, notable differences were observed among the measurement methods. Additionally, differences in solubility were observed between pulse proteins (e.g., chickpea, pea, lentil) and soy proteins, highlighting the impact of amino acid composition on solubility behavior. The Bradford and Lowry methods proved most accurate for measuring solubility in native legume proteins, whereas the Lowry method emerged as the sole viable option for assessing hydrolysate solubility. These findings underscore the importance of selecting appropriate solubility measurement methods, especially in the context of plant-based protein analysis, to ensure accuracy and comparability of results across studies. Standardizing methods in future research can enhance the reliability and confidence in the findings within the plant-based protein field such as gels. Protein gelation plays a direct role in shaping the texture, stability, and sensory attributes of diverse products, thereby enriching the overall eating experience. Additionally, the pH level of the solution can influence the charge and solubility of proteins, thereby affecting their capacity to interact and create gel structures

## 4. Materials and Methods

Pea, chickpea, and soy lentil commercial flours were purchased from local stores. All other chemicals were purchased from VWR (Radnor, PA, USA), Sigma Aldrich (St. Louis, MO, USA), and Fisher Scientific (Waltham, MA, USA), and were of reagent grade.

### 4.1. Protein Purification

#### 4.1.1. Defatting of Legume Flours

The fat from the pea, chickpea, soy, and lentil flours was extracted using two extractions of hexane (Fisher Scientific, Waltham, MA, USA) in a 1:3 ratio. The flour was stirred in hexane for 2 h at 300 rpm using a Caframo Constant-Torque Brushless Mixer (Wiarton, ON, USA) with a Caframo Pitched Blade Impeller 1.5-inch diameter (Wiarton, ON, USA).

#### 4.1.2. Protein Isolation from Defatted Flours

The alkaline extraction and isoelectric precipitation method was modified from Boye et al. [20]. First, 100 g of defatted pea, lentil, chickpea, or soy flour was dispersed in DI water (1:15 ratio for pea flour and 1:10 ratio for lentil, chickpea, and soy). The pH was brought up to 9 using 6.0 N NaOH (VWR Chemicals, Radnor, PA, USA), and then stirred for 1 h at 25 °C. The dispersion was centrifuged at 4500× *g* for 20 min at 4 °C in a Sorvall Legend XFR Centrifuge (Thermo Scientific, Waltham, MA, USA). The supernatant was collected, and the pellet was rehydrated up to the same initial volume and the process repeated to collect the supernatant.

For the isoelectric precipitation, the supernatant from the extraction was brought down to pH 4.5 using 6.0 N HCl (VWR Chemicals, Radnor, PA, USA) and let stir for 30 min. Then, this dispersion was centrifuged at the same settings, and the pellet was collected. The pellet was resolubilized in the same starting mass of water and flour. The pellet and water were homogenized at 3000 rpm for 2 min using an IKA T25 easy clean control ULTRATURRAX homogenizer (Staufen, Germany), brought up to pH 9, held for 5 min, and then brought back down to pH 4.5. The dispersion was again centrifuged, the pellet was collected, and the process was repeated. Then, after centrifugation, the pellet was resolubilized in four times the starting flour weight of water. The dispersion was homogenized at 3000 rpm for 2 min, and the pH was brought to pH 7. The protein was frozen in a −80 °C freezer for 24 h and then freeze-dried. In the rest of this manuscript, pea, lentil, chickpea, and soy proteins are labeled as PPI, LPI, CPI, and SPI, respectively.

#### 4.1.3. Hydrolysis of Protein Isolates

The hydrolysis protocol was modeled after the work of Ghribi et al. with adjustments [9,16]. Freeze-dried protein isolate was suspended in deionized water at 5% *w/w* and allowed to solubilize at 50 °C in a water bath, and pH was adjusted to 7 for Flavourzyme hydrolysis and 8 for Alcalase. Enzyme was added at 1% by mass of protein. Hydrolysis proceeded for 120 min in a gently shaking water bath at 50 °C. Samples were then transferred to a 75 °C water bath for 20 min in order to inactivate the proteolytic enzymes. Samples were then frozen at −80 °C and freeze-dried on a benchtop freeze dryer. An unhydrolyzed protein control was also made using identical processing but without the addition of enzyme. Chickpea–Alcalase, Chickpea–Flavourzyme, Soy–Alcalase, and Soy–Flavourzyme hydrolysates are labeled CPHA, CPHF, SPHA, and SPHF, respectively.

### 4.2. Protein Solubility Methods

#### 4.2.1. Protein Solubility Sample Preparation

For each protein type (soy, lentil, pea, and chickpea), five dispersions of 1% protein isolate concentration were made in DI water. The protein dispersions were stirred for 30 min. Then, the pH was adjusted to 2, 4, 5, 6, or 8. Next, 1 mL of the protein solution was transferred to a microcentrifuge tube in triplicate and centrifuged for 10 min at 4400× *g*. Supernatants from centrifuged protein samples were then diluted and used for protein concentration measurement as specified in those methods. For all the tested methods, solubility is expressed as the percentage of soluble protein in the dry protein isolate samples, calculated as (Mass soluble protein/Mass protein isolate) × 100%.

#### 4.2.2. Bradford Protein Solubility

The Bradford assay method was adapted from the work of Bradford in 1976 used in the literatures [6,16]. Protein solutions were made at 1% *w*/*v* by diluting supernatant 10× in deionized water; then, 50 μL of the diluted supernatant was added to 2.5 mL of Coomassie reagent, incubated for 10 min, and then absorbance was measured at 595 nm using a spectrophotometer.

#### 4.2.3. Biuret Protein Solubility

The biuret method was adapted from the work of Morr et al. [32]. To measure the protein solubility of the samples, 50 µL of the supernatant was transferred to 2.5 mL of biuret reagent. Then, the samples were left to sit for 30 min in the dark. The absorbance was measured at 540 nm.

#### 4.2.4. Lowry Protein Solubility

The Lowry method protocol was adapted from the work of Waterborg and Matthews (1994) [16]. Protein supernatants were diluted 10× and hydrolyzed in 2 N NaOH for 10 min at 100 °C before the addition of complex forming and the Folin–Ciocalteu phenol reagents. Absorbance was measured at 500 nm.

#### 4.2.5. BCA Protein Solubility

The Pierce™ BCA Protein Assay Kit (Thermo Scientific, Waltham, MA, USA) was used. The working reagent was prepared per the instructions from the kit, and then a 1:10 dilution of the supernatant from the protein centrifuge tubes was made for each pH and protein type. Then, 25 μL of the standard or sample was pipetted into a microplate well, and then 200 μL of the working reagent was pipetted and mixed into each well. The microplate was incubated at 37 °C for 30 min, and then cooled to room temperature (25 °C). The microplate was read in a SpectraMax Microplate Reader (Molecular Devices, San Jose, CA, USA) at 562 nm.

Protein concentration was calculated using a BSA standard curve of concentration from 0 to 1.5 mg/mg for all solubility methods. The solubility was calculated by dividing the protein concentration in the supernatant by the total protein in the starting dispersion.

#### 4.2.6. Experimental Design and Statistical Analysis

All conditions were assessed in three separate batches, with two analytical replicates per batch, which resulted in a total number of 6 measurements. Statistical differences in solubility values between methods were analyzed using analysis of variance (ANOVA) with Tukey’s post hoc test, at a significance level of 0.05 using Prism GraphPad Software version 6.

## Figures and Tables

**Table 1:** Average solubility values and standard deviation for soy (SPI), chickpea (CPI), pea (PPI), and lentil (LPI) protein isolates at a range of pHs measured using the Bradford, Lowry, BCA, and biuret methods.

Protein	Method	pH				
		2	4	5	6	8
SPI	Bradford	80.1 ± 1.7 ^a^	1.8 ± 0.5 ^a^	0.9 ± 0.1 ^a^	76.2 ± 2.8 ^a^	82.4 ± 2.2 ^a^
	Lowry	85.0 ± 2.2 ^a^	0.6 ± 0.2 ^a^	0 ± 0 ^a^	81.6 ± 0.7 ^b^	84.6 ± 1.9 ^b^
	BCA	67.3 ± 1.5 ^b^	0 ± 0 ^a^	0 ± 0 ^a^	66.0 ± 1.9 ^c^	68.7 ± 1.1 ^b^
	Biuret	53.6 ± 0.6 ^c^	23.8 ± 0.5 ^b^	13.6 ± 0.2 ^b^	49.7 ± 0.6 ^d^	54.8 ± 0.9 ^b^
CPI	Bradford	85.1 ± 0.4 ^a^	3.9 ± 0.1 ^a^	2.7 ± 0.1 ^a^	47.6 ± 8.7 ^a^	86.3 ± 1.4 ^a^
	Lowry	74.2 ± 1.3 ^b^	6.0 ± 0.9 ^a^	0 ± 0 ^a^	39.0 ± 3.0 ^b^	75.8 ± 1.0 ^b^
	BCA	57.3 ± 0.6 ^c^	0.4 ± 0.2 ^a^	0 ± 0 ^a^	31.2 ± 0.6 ^bc^	57.8 ± 0.8 ^c^
	Biuret	53.2 ± 0.3 ^c^	17.7 ± 0.5 ^b^	13.4 ± 0 ^b^	29.1 ± 3.2 ^c^	53.6 ± 0.4 ^c^
PPI	Bradford	78.4 ± 1.3 ^a^	0 ± 0 ^a^	0 ± 0 ^a^	36.4 ± 0.5 ^a^	77.9 ± 2.8 ^a^
	Lowry	67.6 ± 1.5 ^a^	7.5 ± 0.8 ^b^	0 ± 0 ^a^	36.3 ± 0.9 ^a^	70.3 ± 1.3 ^b^
	BCA	53.3 ± 2.2 ^b^	0.7 ± 0.1 ^a^	0 ± 0 ^a^	19.4 ± 1.2 ^b^	52.9 ± 1.9 ^c^
	Biuret	49.8 ±0.7 ^b^	16.1 ± 0.2 ^c^	13.6 ± 0.2 ^b^	20.4 ± 2.2 ^b^	51.3 ± 1.1 ^c^
LPI	Bradford	82.0 ± 0.8 ^a^	2.9 ± 1.3 ^a^	0 ± 0 ^a^	33.4 ± 1.2 ^a^	82.8 ± 1.1 ^a^
	Lowry	66.9 ± 0.6 ^b^	1.9 ± 0.3 ^a^	0 ± 0 ^a^	35.8 ± 0.1 ^a^	66.9 ± 1.0 ^b^
	BCA	59.5 ± 1.5 ^c^	6.0 ± 1.1 ^a^	0.3 ± 0.4 ^a^	22.0 ± 2.2 ^b^	58.4 ± 2.2 ^c^
	Biuret	52.0 ± 0.5 ^d^	23.4 ± 2.4 ^b^	12.4 ± 0 ^b^	21.3 ± 1.8 ^b^	54.5 ± 0.9 ^c^

Different lowercase letters (e.g., a, b, c) in a column indicate statistical differences (*p* < 0.05) between solubility values for a given method at each pH level for each protein.

**Table 2:** Average solubility values and standard deviation for soy–Alcalase (SPHA), soy–Flavourzyme (SPHF), chickpea–Alcalase (CPHA), and chickpea–Flavourzyme (CPHF) protein hydrolysates at a range of pHs measured using the Bradford, Lowry, and BCA methods.

Protein	Method	pH				
		2	4	5	6	8
SPHA	Bradford	13.9 ± 0.2 ^a^	0.6 ±0.1 ^a^	1.0 ± 0.1 ^a^	3.3 ± 0.8 ^a^	12.1 ± 0.3 ^a^
	Lowry	68.9 ± 1.5 ^b^	51.3 ± 1.1 ^b^	54.4 ± 0.7 ^b^	56.2 ± 1.4 ^b^	67.6 ± 1.0 ^b^
	BCA	36.6 ± 0.7 ^c^	23.2 ± 0.3 ^c^	25.0 ± 0.1 ^c^	27.7 ± 0.2 ^c^	35.2 ± 0.4 ^c^
SPHF	Bradford	51.0 ± 0.9 ^a^	0.8 ± 0.2 ^a^	1.0 ± 0.2 ^a^	31.8 ± 2.5 ^a^	49.9 ± 1.3 ^a^
	Lowry	72.4 ± 3.6 ^b^	19.1 ± 1.6 ^b^	21.5 ± 1.4 ^b^	48.3 ± 3.1 ^b^	73.7 ± 1.9 ^b^
	BCA	52.2 ± 1.4 ^a^	12.4 ± 0.3 ^b^	12.6 ± 0.1 ^c^	29.4 ± 3.6 ^a^	53.4 ± 1.5 ^a^
CPHA	Bradford	8.5 ± 0.7 ^a^	0.6 ± 0.1 ^a^	1.4 ± 0.1 ^a^	4.3 ± 0.4 ^a^	15.6 ± 0.2 ^a^
	Lowry	62.0 ± 1.5 ^b^	43.6 ± 0.6 ^b^	46.1 ± 1.3 ^b^	51.5 ± 1.4 ^b^	63.6 ± 2.0 ^b^
	BCA	29.8 ± 1.0 ^c^	18.1 ± 0.5 ^c^	19.1 ± 0.4 ^c^	20.3 ± 0.2 ^c^	27.5 ± 0.9 ^c^
CPHF	Bradford	29.8 ± 0.6 ^a^	0.1 ± 0.2 ^a^	1.4 ± 0.2 ^a^	11.3 ± 1.1 ^a^	31.2 ± 0.4 ^a^
	Lowry	44.8 ± 0.5 ^b^	19.5 ± 0.4 ^b^	19.2 ± 0.9 ^b^	23.5 ± 1.0 ^b^	44.1 ± 1.1 ^b^
	BCA	29.6 ± 0.6 ^a^	11.4 ± 0.3 ^c^	13.5 ± 1.2 ^c^	16.6 ± 2.0 ^c^	28.3 ± 1.1 ^a^

In a column, different lowercase letters (e.g., a, b, c) signify statistically significant differences (*p* < 0.05) between solubility values for a given solubility method at each pH level for each protein.

## Data Availability

The original contributions presented in the study are included in the article, further inquiries can be directed to the corresponding author.
